# Trends in prevalence of overweight and obesity among South African and European adolescents: a comparative outlook

**DOI:** 10.1186/s12889-022-14724-2

**Published:** 2022-12-06

**Authors:** Emmanuel Nwosu, Anne-Siri Fismen, Arnfinn Helleve, Charles Hongoro, Ronel Sewpaul, Priscilla Reddy, Olufunke Alaba, Janetta Harbron

**Affiliations:** 1 Research Centre for Health Through Physical Activity, Lifestyle and Sport (HPALS), Division of Physiological Sciences, Human Biology Department, Faculty of Health Sciences, University of Cape Town, Anzio Road, Observatory, Cape Town, 7935 South Africa; 2 Department of Health Promotion, Norwegian Institute of Public Health, Bergen, Norway; 3 Centre for Evaluation of Public Health Measures, Norwegian Institute of Public Health, Oslo, Norway; 4 Developmental, Capable and Ethical State Division, Human Sciences Research Council (HSRC), Pretoria, 0001 Gauteng Province South Africa; 5 Health & Wellbeing, Human & Social Capabilities Division (HSC), Human Sciences Research Council (HSRC), Private Bag X9182, Cape Town, 8000, 116 - 118 Merchant House, Buitengracht Street, 8001 Cape Town, South Africa; 6 School of Education, University of KwaZulu-Natal, Durban, South Africa; 7 Health Economics Unit, School of Public Health and Family Medicine, University of Cape Town, Cape Town, South Africa

**Keywords:** Adolescents, Obesity, Overweight, South Africa, Trends in prevalence, Europe

## Abstract

**Background:**

South Africa has several national surveys with body weight-related data, but they are not conducted regularly. Hence, data on longitudinal trends and the recent prevalence of adolescent obesity are not readily available for both national and international reporting and use. This study collectively analysed nationally representative surveys over nearly 2 decades to investigate trends in prevalence of adolescent obesity in South Africa. Furthermore, it compared these data with similar continental report for 45 countries across Europe and North America including United Kingdom, Norway, Netherland, Sweden, Azerbaijan, etc. to identify at-risk sub-population for overweight and obesity among adolescents.

**Methods:**

The study included primary data of adolescents (15 – 19 years) from South African national surveys (*N* = 27, 884; girls = 51.42%) conducted between 1998 and 2016. Adolescents’ data extracted include measured weight, height, sex, parent employment status, monthly allowance received, and family socioeconomic-related variables. Data were statistically analysed and visualized using chi-square of trends, Wald statistics, odds ratio and trend plots, and compared to findings from European survey report (*N* = 71, 942; girls = 51.23%). South African adolescents’ obesity and overweight data were categorized based on World Health Organization (WHO)’s growth chart and compared by sex to European cohort and by family socioeconomic status.

**Results:**

By 2016, 21.56% of South African adolescents were either obese or overweight, similar to the 21% prevalence reported in 2018 among European adolescents. Girls in South Africa showed higher trends for obesity and overweight compared to boys, different from Europe where, higher trends were reported among boys. South African Adolescents from upper socioeconomic families showed greater trends in prevalence of overweight and obesity than adolescents from medium and lower socioeconomic families. Mothers’ employment status was significantly associated with adolescents' overweight and obesity.

**Conclusions:**

Our study shows that by 2016, the prevalence of adolescent obesity was high in South Africa – more than 1 in 5 adolescents – which is nearly similar to that in Europe, yet South African girls may be at a greater odd for overweight and obesity in contrast to Europe, as well as adolescents from high earning families. South African local and contextual factors may be driving higher prevalence in specific sub-population. Our study also shows the need for frequent health-related data collection and tracking of adolescents’ health in South Africa.

## Background

According to the World Health Organization (WHO) [1], obesity is now a global public health concern. It is estimated that about 340 million children and adolescents (5 -19 years old) are either obese or overweight. From as low as 4% in 1975, pediatric obesity and overweight rose to 18% by 2016, with 18% of girls and 19% of boys being overweight, and 6% of girls and 8% of boys being obese worldwide [1]. Adolescents are of concern because obesity at this stage could lead to premature death, and disabilities in adulthood. Furthermore, as they grow into adults, health conditions associated with overweight including chronic cardiovascular conditions like hypertension will be more severe [2, 3]. There are also existing psychosocial impacts like stigma and discrimination [4, 5] associated with adolescent obesity.

Obesity was previously seen as a problem of high-income countries (HIC) but, data from the past decade show that low- and middle-income countries (LMICs), especially in sub-Saharan Africa, bear the double burden of underweight and overweight [6–8]. Hence, with the shift in the global burden of adolescent overweight and obesity, current prevalence and trends are required for South Africa, a middle-income, sub-Saharan African country that is experiencing sweeping industrialization and urbanization. Furthermore, the roles of sex and family socioeconomic status (SES) are not clearly defined in South African adolescent obesity and overweight trends.

As LMICs become more industrialized, they are likely to acquire both the benefits and problems associated with industrialization which include obesity [9]. South Africa (SA) as a LMIC is undergoing a socioeconomic transition related to urbanization and industrialization in most of its cities with a corresponding energy imbalance due to consumption of highly processed and calorie-dense diets, lack of built structures that encourage being physically active, etc. This nutritional transition is closely linked with obesity transition.

The obesity transition model [10] describes global trends in relation to population parameters and determinants that predict the trajectory of obesity and future outcomes based on extensive tracking of available data. Most Sub-Saharan African and South Asian countries are believed to be in the first stage of the transition model, characterised by higher obesity prevalence in women and those with a higher SES among adults [10]. Furthermore, European countries are said to be in the third stage, characterized by higher obesity prevalence among those in lower SES, and a plateau in prevalence among women in high SES and children [10]. A comparison between SA and European adolescent obesity data will facilitate an understanding of the main local and contextual drivers of these trends and prevalence and furthermore, in developing policies for its mitigation.

Surveillance data on adolescents overweight and obesity are not regularly collected in SA as done in HICs like in Europe. Although there are several nationally representative surveys conducted at different times over the past 2 decades in South Africa, which reported on the prevalence among South Africans of overweight and obesity, none of them were conducted regularly. Hence, these surveys need to be collectively analysed to investigate prevalence and determine trends to date. In addition, the association of sex and family SES with these trends and prevalence over the past 2 decades requires full investigation.

Hence, this study aims to investigate South African adolescents’ obesity and overweight trends in prevalence by collating, analysing, and generating trend patterns with data from national surveys. In addition, it aims to investigate and report the sub-populations at greater risk of adolescent overweight and obesity based on sex and family socioeconomic measures. Furthermore, to compare South African and European trends and identify contextual and local factors related to the obesity transition conceptual model [10].

These will facilitate developing context-relevant strategies and policies for mitigation and prevention of adolescent obesity and overweight in SA and sub-Saharan countries.

## Methods

The study is an exploratory and quantitative analysis of primary data collected from three national South African (SA) surveys and compared to a continental European survey report to investigate trends in prevalence of overweight and obesity. SA Survey data were independently collected, and analyses were performed on the pooled samples to draw trend-related inferences.

### Data sources

The South African surveys and the year of data collection are as follows:

- **National Youth Risk Behavior Surveys (YRBS) 2002, 2008 and 2011** [8, 11]
- **SA National Health and Nutrition Examination Survey – SANHANES – 1 **[12].
- **SA Demographic and Health Surveys (SA DHS) –** https://dhsprogram.com/; [12].

SA DHS was conducted 3 times – 1998, 2003 and 2016 but 2003 data was removed from the public domain because of concerns about its quality, hence this study extracted and analysed data only from 1998 and 2016 for which the data qualities are acceptable and are in the public domain.

We further reported findings from the European survey report –

- **Health Behaviour in School-aged Children (HBSC) survey—2010, 2014 and 2018** (http://www.hbsc.org). The HBSC survey is conducted every fourth year and survey years included are the most recent and comparable years with South African surveys. These survey years applied similar World Health Organization (WHO) growth chart as used in the South African cohort for the classification of overweight and obesity, to aid in the comparison of the two cohorts [13, 14].

Primary data of participants aged 15–19 years were extracted from SA surveys and analysed. The total sample size of SA participants across the 3 surveys used for the study (N) is 27 884 (Girls: 51.42%, (mean ± standard deviation (sd)) age: 16.83 ± 1 years). In this study, we present findings based on the HBSC survey published report for participants aged 15 years old (*N* = 71; Girls = 51.23%).

Two of the SA surveys’ sampling methods applied the Statistics South Africa’s census enumeration area (EA) as the primary sampling unit (PSU) – SANHANES and SADHS – while the YRBS used schools as the PSU. All three surveys applied a stratified cluster sampling design as follows; in the YRBS, classes within selected schools were sampled, and all individuals in each selected class were eligible to participate. In SANHANES and SADHS, households within selected EAs were randomly sampled and individuals within selected households were eligible to participate. The findings from the European survey were based on the published summary report [14] hence HBSC data were not analysed in the current study.

The key measures of interest for this study are as follows:

### Overweight and obesity

The SA overweight and obesity data are based on measured weight and height collected according to a standardized protocol for all three surveys [8, 11, 12, 15]. The European report is based on young people’s self-reported height (without shoes) and weight (without clothes). In this study, we computed BMI as weight divided by height squared. BMI was categorised according to the World Health Organization (WHO) BMI-for age Z-score (BAZ) classification for age and gender [16]. Cut-offs for overweight and obesity were allocated based on the WHO growth reference chart for age and gender. Similar WHO growth reference for age and gender was also used for the classification of the HBSC survey as reported in the protocol [13].

### Socio-economic status (SES)

For the SA data, different SES variables related to adolescents’ family wealth as measured in the SA setting and as recommended in previous studies were used [17, 18]. In the SANHANES (2012) and SADHS (1998 and 2016) surveys, variables of interest are household possessions owned by adolescents’ families, specifically refrigerator and washing machine, source of drinking water, type of residential house and type of toilet, type of cooking fuel used by the family and presence or absence of electricity supply for the family. These variables were used to compute wealth indices of study participants’ families using the principal components analyses (PCA) method [19, 20]. Further, the wealth index values were categorised into one of the following 3 groups – lower, medium and upper to make it comparable to HBSC data [13] and increase statistical power of each of the wealth index groups following the international DHS format [19, 20].

For the YRBS data of 2002, 2008 and 2011, SES was assessed using parents’ employment status (father and mother) as well as the amount received by adolescents as monthly allowance, in SA Rand (ZAR). A dichotomous variable indicating if their father and mother are “employed” or “unemployed” was created for parents’ employment status as well as a variable indicating if “none”, “one” or “both” parents are employed. These indicated the cumulative earning ability of the parents (income). For the monthly allowance received by adolescents, participants were placed in one of 3 groups as follows lower (2002: None – ZAR 20; 2008 & 2011: None – ZAR 40), medium (2002: ZAR 21 – 50; 2008 & 2011: ZAR 41 – 70) and upper (2002: > ZAR 50; 2008 & 2011: > ZAR 70). The classification is based on the monthly reported allowance amount and inflation rate at each survey year. The exact questions that assessed parents’ employment status and adolescents’ monthly allowances are as follows.

***Father***: “*Does your father have a paid job? (paid job also refers to those who are self-employed e.g. your father has a shop at home)*”

***Mother***: “*Does your mother have a paid job? (paid job also refers to those who are self-employed e.g. your mother has a shop at home)*”

***Monthly allowance***: “*In a normal / usual month (30 days), how much spending money do you get?*“

The empirical results about SA participants’ families’ socioeconomic measures were compared to narrative findings presented in the HBSC report

### Statistical data analyses

To investigate overweight and obesity proportion and trends in prevalence in the SA adolescents’ cohort, extracted weight and height of participants were imported to STATA software (https://www.stata.com/) with adolescents’ sex and age (in months) variables. Body Mass Index (BMI)-for-age Z-score (BAZ) was computed using the WHO STATA macros algorithm (https://www.who.int/tools/growth-reference-data-for-5to19-years/application-tools) for all surveys. Participants whose BAZ met WHO’s recommended category for Overweight (BAZ >  + 1 SD and ≤  + 2 SD) and Obesity (BAZ >  + 2 SD) in all the surveys were included in computing the proportion of adolescents’ overweight and obesity in the subsequent analyses for each year and to generate trend plots, frequency distribution and bar plots. We report proportion and trend in prevalence results with time. The significance of trends was assessed using the chi-square test for trend (*X*^*2Trend*^). All data were reported at a significance level of *p* < 0.05. In addition, trends in prevalence for overweight and obesity were assessed based on adolescents’ sex (male and female) and socioeconomic status (SES – wealth indices, parents’ employment status and monthly allowances received by adolescents) measures. Trend plots of overweight and obesity were also compared by adolescents’ sex between South African and European data. We also assessed the effects and significance of effects (*p* < 0.005) for adolescents’ sex and family SES measures on overweight and obesity using odds ratio and Wald test statistics, and reported odds ratio by year.

## Results

### Sample characteristics

Table 1 shows sample demographics for adolescent study participants from the three SA surveys extracted for this study. For all surveys, the number of boys and girls were comparable as indicated by similar percentage proportion. The mean BMIs for all survey years are within 21.45 – 22.40 kg/m^2^. Adolescents’ families in the lower socioeconomic group classified based on the wealth index are in greater proportion (81.59%) than medium (14.25%) and upper (4.16) groups for survey year 2012 (Table 1). The proportions of fathers and mothers who are employed are nearly equal in proportion to those who are not, while the disparity between both parents being employed and not, was lowest for survey year 2008 (Employed – 27.65%; Unemployed – 26.90%).

**Table 1:** Socio-demographic and socio-economic status information of participants (aged 15—19 years) from South African surveys

South African surveys						
	SADHS 1998	YRBS 2002	YRBS 2008	YRBS 2011	SANHANES 2012	SADHS 2016
***Sample size (N)***	2143	6915	8243	8296	1081	1206
***Male; n (%)***	1067 (49.79)	3367 (48.69)	4128 (50.08)	3923 (47.29)	466 (43.11)	595 (49.34)
Mean (*sd) age (years)	17 (1)	17 (1)	16 (1)	17 (1)	17 (1)	17 (1)
Mean (*sd) Body Mass Index (BMI – kg/m^***2***^***)***	21.45 (4.02)	21.48 (4.47)	21.65 (4.64)	21.75 (5.58)	22.40 (5.09)	22.06(4.38)
*Socioeconomic measures*						
*Participants’ families’ wealth Index*						
**Lower n (%)**	696 (32.48)				882 (81.59)	568 (47.10)
**Medium n (%)**	643 (30.01)				154 (14.25)	265 (21.97)
**Higher n (%)**	804 (37.52)				45 (4.16)	373 (30.93)
***Father Employed (%)***		47.94	47.91	47.11		
***Mother Employed (%)***		42.51	43.45	43.57		
***Number of participants with both Parents employed / unemployed (%)***		24.76/29.99	27.65/26.90	32.95/27.27		
*Number of participants receiving Monthly allowance by groups (n)*						
**Lower**						
*2002:* None – ZAR 20 *2008 & 2011:* None – ZAR 40		62.78	52.93	51.53		
**Medium**						
*2002:* ZAR 21 – 50 *2008 & 2011:* ZAR 41 – 70		19.37	20.76	18.77		
**Upper**						
*2002:* > ZAR 50 *2008 & 2011:* > ZAR 70		17.85	26.32	29.69		

*SADHS* South Africa Demographic and Health Surveys, *YRBS* National Youth Risk Behavior Surveys, *SANHANES* South Africa National Health and Nutrition Examination Survey, *BMI* Body Mass Index, *sd* standard deviation

### Overweight and obesity prevalence

**Fig. 1:**
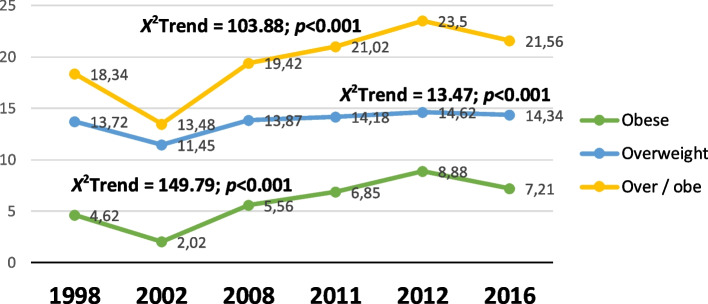
Obesity and overweight trends in prevalence for South African adolescents. Legend: Percentage proportion (%) of trends in prevalence for South African adolescents (15 – 19 years) who are overweight or obese from 1998 – 2016. *Over/obe – Overweight – obese

Within the SA cohort, inspite of an initial reduction in proportion (from 18.34% to 13.48%) by 2002, there was a gradual and consistent increase in the proportion of adolescents who were overweight or obese until 2012 (Fig. 1). This appears to be driven by an increase in the proportion of the obese group. By 2016, the proportion of adolescents who were overweight or obese was 21.56% and nearly equal to the Europe adolescents’ proportion (21%) who were either overweight or obese in 2018. All changes in trends were significant (*p* < 0.001) with time.

### Overweight / obesity by sex

**Fig. 2:**
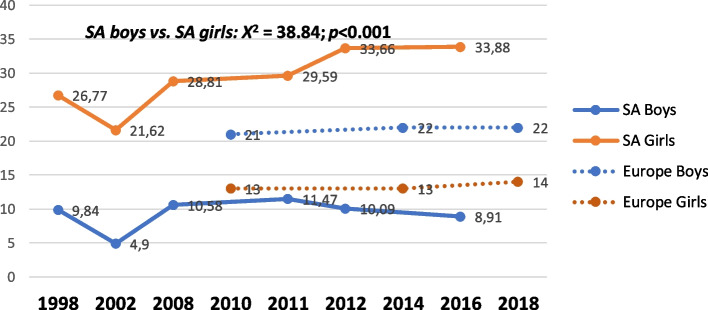
Obesity and overweight trend in prevalence by sex. Legend: Percentage proportion (%) of overweight-obesity trend for South African adolescents (15 – 19 years) in 1998, 2012 and 2016 by sex

Across the survey years, our results showed that boys consistently had lower prevalence of overweight and obesity than girls and this difference is statistically significant (Fig. 2). From 1998 to 2011, SA boys and girls followed a similar trend in overweight-obesity proportion. Subsequently, a reduction in the percentage of boys’ who are overweight or obese was observed until 2016 (8.91%), while the trend for girls increased further to 33.88% (Fig. 2). Wald Chi-square (*X*^*2*^) showed a significant association between sex and adolescents’ overweight and for all the years assessed – 1998, 2002, 2008, 2011 and 2012 – except in 2016 (Table 2). Odds ratio (OR) result showed that by 2016, there was 1.13 higher odds of a girl becoming obese or overweight than a boy in South Africa.

**Table 2:** Odds ratio by year indicating Sex effect on overweight and obesity in South African adolescents

SURVEY YEAR	ODDS RATIO (OR)	95% CONFIDENCE INTERVAL
**1998**	2.91*	1.87, 4.68
**2002**	0.60*	0.42, 0.84
**2008**	0.38*	0.31, 0.46
**2011**	0.39*	0.32, 0.48
**2012**	4.56*	2.67, 8.33
**2016**	1.13	0.70, 1.82

^*^Sex effect significant at *p* < 0.05 (Wald Chi-square (*X*^*2*^), base category as girls vs. boys

In HBSC findings, a greater proportion of boys were overweight or obese from 2010 to 2018, when compared to girls, in contrast with SA where a greater proportion of the cohort who were overweight or obese was girls (Fig. 2). Furthermore, there was almost no change with time in Europe’s percentage of adolescents who were overweight or obese compared with their SA counterparts, where there was a considerable change with time.

### Obesity / overweight by socioeconomic groups

**Fig. 3:**
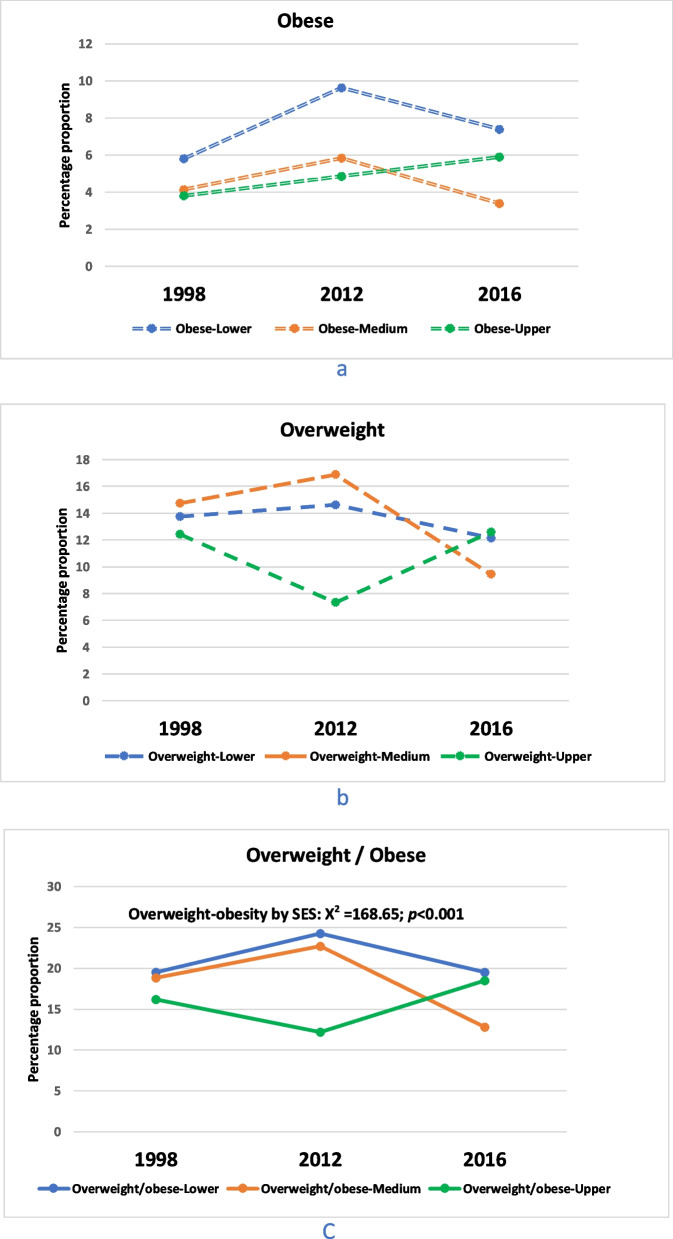
Trends in prevalence of obesity and overweight by Wealth index. Legend: Percentage proportion (%) of overweight-obesity trend for South African adolescents (15 – 19 years) in 1998, 2012 and 2016 by family socioeconomic status (SES) measured by Wealth Indices

SA wealth index data (Fig. 3) from 1998, 2012 and 2016 showed that obesity prevalence among adolescents from families with a medium SES was the highest at all three-time points (Fig. 3a). However, the trends indicated that obesity prevalence in adolescents from families with higher SES increased with time, in contrast with medium and lower SES groups, and peaked at 5.90% in 2016 (Fig. 3a). Furthermore, adolescents from medium and lower SES families followed a similar trajectory that showed decrease in overweight and obesity proportion recently, which is different from adolescents from upper SES whose proportions have increased recently (Fig. 3c). The differences in the proportion of adolescents who were overweight or obese with time from the three SES groups were statistically significant (Fig. 3c).

**Fig. 4:**
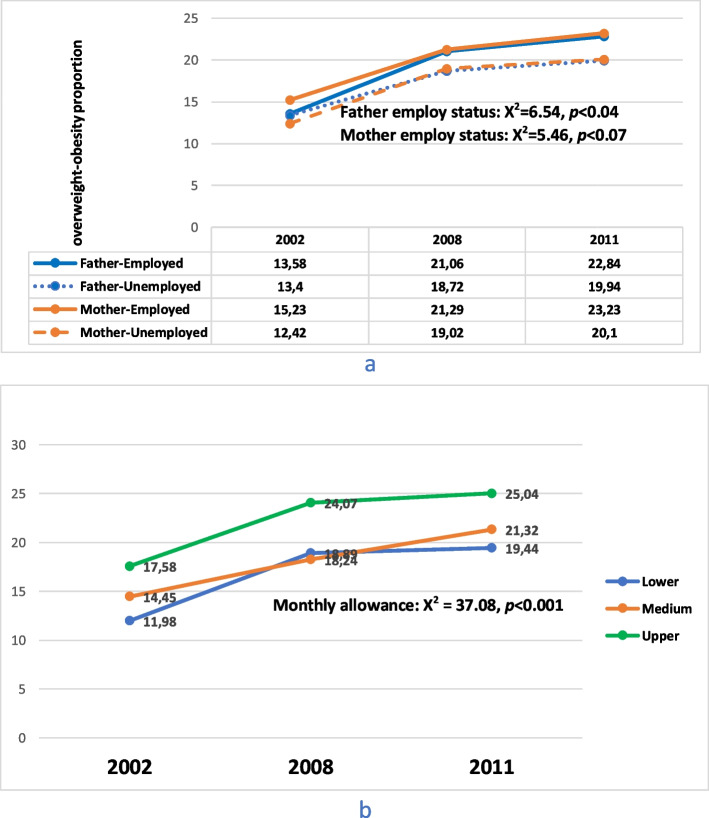
Obesity and overweight trend by parents’ employment status and monthly allowance received. Legend: Percentage proportion (%) of overweight and obesity trend for South African adolescents (15 – 19 years) in 2002, 2008 and 2011 by **a**) Father and Mother employment status, **b**) monthly allowances received

Data from the 2002, 2008 and 2011 YRBS showed that the proportion of adolescents who were overweight or obese and with employed parents was slightly higher than those whose parents were unemployed (Fig. 4a). The difference in proportion of adolescents who are overweight or obese with either employed or unemployed fathers was significant (x^2^ = 6.54, *p* = 0.04) but not for mothers. Figure 4b, also showed that the number of overweight or obese adolescents who received higher monthly allowance (upper group) was greater compared to those in the medium and lower groups. The difference in proportion between the three groups was significant (x^2^ = 37.08, *p* < 0.001) with time.

Table 3 showed that family wealth indices for lower and medium SES families were significantly associated (OR – 0.44; 95% CI – 0.20, 0.88, *p* = 0.03) with adolescents’ overweight or obesity only in 2016, but monthly allowance of medium and upper groups of adolescents was significantly associated with either being overweight or obese in 2008 (OR – 0.69; 95% CI – 0.55, 0.85, *p* < 0.001) and 2011 (OR – 0.69; 95% CI – 0.57, 0.84, *p* < 0.001). Furthermore, fathers’ employment status was not associated with being overweight or obese, but mothers’ employment status had a significant association with adolescents being either overweight or obese in 2002, 2008, 2011 (Table 3).

**Table 3:** Socioeconomic measures effects on overweight-obesity proportion assessed with odds ratio by Year

	**Odds ratio (OR)**	**95% Confidence interval**
**Socioeconomic measure by Wealth index**		
**1998**		
*Lower vs. Medium*	0.70	0.42, 1.14
*Medium vs. Upper*	1.08	0.64, 1.83
**2012**		
*Lower vs. Medium*	0.58	0.27, 1.12
*Medium vs. Upper*	1.21	0.30, 8.15
**2016**		
*Lower vs. Medium*	0.44*	0.20, 0.88
*Medium vs. Upper*	0.56	0.24, 1.20
**Socioeconomic measure by Adolescents’ monthly allowance**		
**2002**		
*Lower vs. Medium*	0.73	0.48, 1.13
*Lower vs. Upper*	0.65	0.43, 1.00
**2008**		
*Lower vs. Medium*	0.99	0.77, 1.29
*Lower vs. Upper*	0.69*	0.55, 0.85
**2011**		
*Lower vs. Medium*	1.01	0.80, 1.30
*Lower vs. Upper*	0.69*	0.57, 0.84
**Parent Employment Status**		
*Father*		
**2002**	0.85	0.57, 1.26
**2008**	1.30	1.02, 1.68
**2011**	1.02	0.82, 1.27
*Mother*		
**2002**	1.57*	1.11, 2.24
**2008**	1.33*	1.08, 1.65
**2011**	1.26*	1.04, 1.53

^*^Parent employment status effect significant at *p* < 0.05 (Wald Chi-square (*X*^*2*^)

## Discussion

The current study uses the most recent, available, and nationally representative data to investigate the trends in the prevalence of adolescents overweight and obesity in South Africa. The study compared SA adolescents’ overweight and obesity prevalence to a European cohort from a reliable European adolescent survey report (HBSC) and further applied the obesity transition model to predict the sub-populations at greater risk with respect to gender and socioeconomic status. Our findings showed statistically significant increase in the proportion of overweight and obese adolescents from 1998 to 2016 in SA. Furthermore, a similar percentage in the SA cohort was observed in in 2016 when compared to that of Europe in 2018. We also found a significantly greater proportion of girls being either overweight or obese in South Africa in contrast to Europe and further among adolescents from high SES families.

### SA trend in prevalence of adolescent overweight and obesity

Our findings of increasing overweight and obesity with time among SA adolescents confirmed our initial hypothesis and findings from previous studies which showed that obesity prevalence among South African adolescents increased with time [21, 22]. This, as reported by other studies is not unrelated to current urbanization and industrialization sweeping through most LMIC, including South Africa, and the associated nutritional transition from more traditional healthy diets to westernized, refined and highly processed foods [10]. Furthermore, environmental factors encouraging a sedentary lifestyle and other unhealthy dietary behaviours are implicated as major drivers of the increasing prevalence [22, 23].

Our findings also showed that girls and adolescents from high socioeconomic families were at greater risk of being overweight or obese. For example, in 2016, girls were more than 10% more likely to be obese than boys in South Africa. Similarly, Grobbelaar et al. [24] and Otitoola et al. [21] have previously identified girls as having a higher prevalence of overweight and obesity than boys in a child welfare home in Durban, Kwazulu-natal and at Comfivaba, Eastern Cape respectively, both in South Africa. It is also evident from our results that among girls, overweight and obesity prevalence have increased over time but decreased since 2011 among boys. There are suggestions that the risk of overweight and obesity in adolescent girls may be related to the onset of menstruation, its associated hormonal changes and psychological impact on physical activeness. In group sessions, adolescent girls shared their perceptions that teenage pregnancies and contraception use contributed to weight gain [25]. In contrast, adolescent boys at the same age, mark an increase in energy needs and physical activities leading to “burning of fats” and reduced energy storage [22]. Other factors also linked to the boys-girls disparity in overweight are cultural and behavioural factors. For example, in some traditional African cultures being overweight is seen as an indication of being healthy, happy, and not suffering from debilitating conditions like HIV/AIDS [26]. We propose that multiple sociocultural, physiological and behavioural factors increase the odds of overweight and obesity among adolescent girls in the South African setting, hence, to address these factors require multi-pronged strategies and collaboration of local communities, government and private institution.

With regards to adolescents from high SES families within the context of our study sample, the overweight and obesity observed in higher proportion may be because of the tendency for increased consumption of processed and fast-food products among those who have the financial means. Our findings are similar to studies collated in a sub-Saharan African review [27] which showed that children from the highest SES are about 5 times at greater risk of overweight and obesity than those from the lowest SES household. Aounallah-Skhiri et al. [28] showed in a North African study that one of the major outcomes of urbanization and high income is the tendency for consumption of processed food including white bread, dairy products, high-fat content, and high-energy products. We hypothesize that adolescents from higher income bracket in this study are more likely to be able to “purchase” comfort including buying electronic gadgets like smartphones and video games which encourage a sedentary lifestyle and reduce being physically active [29]. Furthermore, they are more likely to have means of affording unhealthy food compared to adolescents from medium and low-income families. This hypothesis is supported by our results that indicated that adolescents with higher monthly allowance had higher overweight obesity prevalence. McLaren [29] and Pavela et al. [30] also indicated that the relationship between obesity and socioeconomic status (SES) is nuanced by several other factors including ethnicity, sex and geographic location. For example, while obesity is associated with high SES in low-income countries, the reverse is the case in high-income countries [29].

We found that mothers’ employment status was significantly associated with the proportion of adolescents who were overweight or obese in each survey year in contrast to fathers’ employment status. This is similar to previous findings in UK and USA [31, 32], both judged as fully industrialized nations, where mothers’ employment was associated with children gaining weight. The pathway for the increase in weight is that mothers’ availability after school is seen to reduce sedentary behaviour among adolescents [32], while mothers’ employment led to poorer eating habits and an increase in sedentary behaviour among children [31]. In the South African context, the impact of traditional gender roles is an important factor to consider. Women are expected to look after the house and ensure that children live a healthy life including preparing meals for the family. We hypothesize that employed mothers are likely to be unavailable and ensure that adolescents engage in activities and behaviours that mitigate overweight and obesity, which could be the reason for the association between mothers’ employment status and adolescents’ overweight and obesity.

### South Africa’s trend in prevalence compared to Europe findings

Contrary to our hypothesis, our findings showed that SA and Europe have almost equal proportion of adolescents with obesity and overweight, although the steeper slope in the gender plot indicates that the rate of progression of the pandemic may be greater in SA. South Africa is an upper -middle-income economy and is seen as one of Africa’s most industrialized and civilized economy, hence we propose that the similarities in most economic indices between South Africa and European nations create a nearly similar sociocultural context including factors affecting adolescents overweight and obesity. Studies have shown that the rate of obesity progression is high in LMIC as in HIC [33]. According to World Health Organization, most LMICs are now equally at risk as HICs of overweight and obesity due to nutritional transition in the urban centres and lack of built and social environments that encourage being physically active [1, 9]. Armstrong et al. [26]’s finding showed that among South African children, overweight and obesity proportion are similar to what was observed in the USA in the previous decade. Hence the need for immediate and aggressive policy actions.

An important finding in this comparative study is the gender disparity in overweight and obesity burden. While South African girls are at greater risk of being overweight or obese compared to boys, the European report presents the exact opposite. These findings may be influenced by disparity and nuanced interaction of cultural emphasis and current social environment in both locations. Awareness of cultural influence and social pressure is important in understanding the South African finding. Most African culture link health and living well to being plump and fat [26, 34]. Females are more desirable and attractive in the African setting when they are not slim and lean, especially in a context where being lean or slim is attributed erroneously to debilitating diseases like HIV and AIDS [22, 26]. Being plump and fat is mostly associated with fertility and therefore, there is social pressure even in the early years of adolescence which could contribute significantly to the proportion of girls who are overweight or obese. In contrast to Africa, most developed countries’ cultures like in Europe link beauty and attraction to being slim and lean [21, 26]. There are indications that social media and peer effects have increased the pressure among girls in developed nations to ensure lean and slim body structure [35, 36]. Some extreme weight loss behaviours (EWLB) associated with this social pressure among girls include irregular fasting and skipping of meals, self-induced vomiting, laxative, and diet pill use [14, 37].

Furthermore, cultural factors may also explain the contrasting findings among boys. While the traditional African culture emphasizes fattening girls in preparation for marriage [22, 26], boys are socialized to be active and to work hard in preparation to be the breadwinner of the family. Hence, most boys are influenced by this culture to be physically more active than their female counterparts, unlike in high-income countries like Europe where there is less social pressure on males to be the sole breadwinner, work harder or engage in more physically taxing work than their female counterparts. Findings show that in Europe, proportion of adolescents engaging in moderate-to-vigorous physical activity decreases from age 11 to 15 years between 2006 and 2018 [14, 38]. Hence fewer boys engaging in physical activity at the age of 15 years would have played an important role in the increasing proportion of European boys being overweight and obese in contrast to the SA cohort.

### South Africa’s overweight and obesity trend in prevalence, and the obesity transition model

Our study showed an increase in the percentage of SA adolescents who were overweight or obese between 1998 and 2016. Although SA adolescent population is generally at risk of overweight and obesity, girls and adolescents from high SES families may be at greater risk. According to the obesity transition model [10], there are four stages in obesity transition. Specifically, and going by our findings it will appear that the SA adolescent population is going through the first stage of the model. At stage one, there is a higher prevalence of obesity in females compared to males, and higher in those from higher SES compared to lower SES [10]. Many countries in South Asia and sub-Saharan Africa are also at this stage [10], which appears to be plausible within the SA adolescent cohort given our findings in this study.

According to Jaack et al. [10], the importance of using the obesity transition model to predict its prevalence is to enable stakeholders including researchers and policymakers to gain guidance in identifying the subpopulation at greater risk and the stage within specific communities in order to take proactive steps to prevent severe prevalence while considering all local contextual influences. The final aim of this study is to shine the spotlight on the at-risk subpopulation for overweight and obesity within the adolescent cohort, specifically girls and adolescents from high SES especially those living with employed mothers. Further study will hopefully elucidate the environmental and behavioural factors that put these subpopulations at risk of overweight and obesity, and possibly raise points of discussion among stakeholders on how to prevent further increase, among the adolescent population as the future workforce in South Africa.

### Study limitation

A limitation of the current study is the imbalance in the proportion of adolescents’ sample size based on wealth index grouping for 2012 survey year. Specifically, the higher SES participants were smaller in number compared to middle and lower SES groups. This could be because the adolescent cohort from the original survey was skewed towards the lower SES families, although all SES groupings were matched equally when other age groups, not relevant in this study, were included. Regardless of this discrepancy, our findings regarding SES link with adolescent overweight and obesity are consistent with other SES measures that didn’t include data from 2012 sample. Furthermore, socioeconomic data for all 3 SA surveys were collected differently, yet they are all nationally representative sample. Finally, it is important to note that all European data used in the study came from the survey report, hence data analyses was not performed on HBSC data in this study. 

## Conclusions

This study is one of the first aimed at using both the obesity transition model and comparative approach to understand the trends in the prevalence of adolescent overweight and obesity in the South African context over a period of about two decades. Then to further identify the at-risk subpopulation in order to provide guidance to stakeholders in mitigating the pandemic. Our findings showed that the prevalence of adolescent overweight and obesity has increased with time until 2016 based on data collected in the national surveys. Furthermore, girls and adolescents from higher socio-economic families, especially those whose mothers are employed, may be at a greater risk for overweight and obesity in our local context. Further investigation on this cohort will highlight behaviours of the at-risk subpopulation that mediate the increased prevalence. This will assist researchers and policymakers in developing policies and strategies to mitigate and prevent further increases in overweight and obesity among the adolescent cohort. Further follow-up and regular adolescent-focused surveys are needed to track adolescents’ health and behaviours. 

## Data Availability

The data that support the findings of this study are available from Statistics South Africa (StatsSA), Human Sciences Research Council (HSRC) South Africa and International Coordinating Centre (ICC) Health and Behaviour in School-aged Children University of Glasgow but restrictions apply to the availability of these data, which were used under license for the current study, and so are not publicly available. Data are however available from the authors upon reasonable request and with permission of Statistics South Africa (StatsSA), Human Sciences Research Council (HSRC) South Africa and International Coordinating Centre (ICC) Health and Behaviour in School-aged Children University of Glasgow.

## Abbreviations

WHO: World Health Organisation
HIC: High-income country
LMIC: Low- and Middle-income country
SES: Socioeconomic Status
SA: South Africa
YRBS: National Youth Risk Behaviour Survey
SANHANES: SA National Health and Nutrition Examination Survey
SA DHS: SA Demographic and Health Surveys
HBSC: Health Behaviour in School-aged Children
EA: Enumeration Area
PSU: Primary Sampling Unit
BMI: Body Mass Index
BAZ: BMI-for age Z-score
PCA: Principal Components Analyses
ZAR: SA Rand
*X*^*2Trend*^: Chi-square test for trend
OR: Odds Ratio
HIV: Human Immunodeficiency Virus
AIDS: Acquired Immune Deficiency Syndrome
UK: United Kingdom
USA: United States of America
